# Distinctiveness of long-term pain that does not interfere with life: An observational cohort study

**DOI:** 10.1002/j.1532-2149.2012.00118.x

**Published:** 2012-02-21

**Authors:** KP Jordan, J Sim, A Moore, M Bernard, J Richardson

**Affiliations:** 1 Arthritis Research UK Primary Care Centre, Primary Care Sciences, Keele UniversityUK; 2 Centre for Social Gerontology, Keele UniversityUK

## Abstract

**Background:**

Reporting of pain that does not interfere with life is common in the older population but little is known about people with such long-term non-interfering pain.

**Objectives:**

To assess whether non-interfering pain can be a long-term state, and to compare this group with those who continuously report no pain, and with those with chronic pain that interferes with life.

**Methods:**

This was a prospective general population cohort study set within the North Staffordshire Osteoarthritis Project (NorStOP). People aged 50 plus were sent baseline, 3-year and 6-year questionnaires. Those who reported the same pain status (no pain, non-interfering pain, interfering pain) at each time point were compared on pain intensity, widespread pain and medication, and on sociodemographic and co-morbid characteristics at 6 years.

**Results:**

Forty percent of responders reported the same pain status at each time point; 12% reported long-term non-interfering pain. Fifty-nine percent of those with non-interfering pain reported at least one site of high pain intensity, 33% reported widespread pain, and 90% had used pain medication in the past 4 weeks. This group was similar to the no-pain group but distinct on sociodemographic and co-morbid measures from those with pain that interfered.

**Conclusions:**

Long-term non-interfering pain is common, but despite often suffering from high pain intensity and widespread pain, those within this group seem to be able to control their pain without allowing it to affect their everyday lives. Future work is needed to assess how people with long-term pain ensure it does not cause interference with life.

## 1. Introduction

Prevalence of self-reported pain appears consistent across age groups in older adults with up to two-thirds reporting pain in a 4-week period (Scudds and Ostbye, 19; Thomas et al., 24). Some types of regional pain, such as back pain, have shown declines in prevalence with age, whereas others increase (Sternbach, 22; Urwin et al., 26; Gibson and Helme, 7; Helme and Gibson, 8; Thomas et al., 24). Onset of interfering pain, defined as pain that interferes with normal daily activities, appears to increase with age (Thomas et al., 23). However, many within the older (50+ years) community-dwelling population remain free from pain, or report no interference from existing pain (Thomas et al., 24; Shi et al., 20).

Pre-existing pain complaints, anxiety, depression, smoking, obesity and age have been linked with onset of interfering pain in older people (Peters et al., 17; Jordan et al., 10; Shi et al., 20). Social factors including inadequate income, neighbourhood deprivation and education are linked to interfering pain (Jordan et al., 10; Shi et al., 20; Dorner et al., 6). It is unknown whether pain status is generally a changing state (moving between interference, non-interference and no pain) or if it can also be a long-term unchanging state. Similarly, it is not known how different those who continuously report non-interfering pain are from those with long-term interfering pain on characteristics linked with the onset of interfering pain (e.g. depression, obesity), or whether these differences are simply due to the severity and widespread nature of the pain. If people with long-term non-interfering pain do have characteristics associated with interfering pain, then this group could be targeted to assess how interfering pain may be prevented despite having these characteristics. However, if there exists a group of people with long-term non-interfering pain who have not developed these characteristics, this suggests that this group should be the focus of research that aims to establish if and how development from non-interfering to long-term interfering pain can be halted or delayed. Ultimately, the distinguishing characteristics identified between those with long-term non-interfering pain and those with interfering pain may then be targeted for the development of strategies to stop progression from non-interfering to long-term interfering pain status.

This paper reports on the first phase of a mixed-methods study on preventing pain from interfering with later life. The main study examines how older people can be helped to maintain daily activities in the presence of pain. The objectives of this phase were to assess whether long-term non-interfering pain is common in the older population, and to compare this group on characteristics associated with interfering pain with those who continuously report no pain, and with those who have chronic pain that interferes with their life. Finally, to assess whether any differences between those with non-interfering pain and those with interfering pain are due to the severity or widespreadness of pain, or whether there is a group who maintains daily activities and with distinct characteristics despite severe and widespread pain.

**What's already known about this topic?**

- Reporting of pain that does not interfere with life is common in the older population but little is known about people with such long-term non-interfering pain.

**What does this study add?**

- Pain that is non-interfering can be a long-term state, characterised by high levels of pain medication and frequently involving widespread pain.
- However, those with non-interfering pain do not have many of the characteristics associated with interfering pain and appear to be able to control the effects of their pain.

## 2. Methods

The study was based within two cohorts of the North Staffordshire Osteoarthritis Project (NorStOP), a prospective cohort study of joint pain and general health in older people (Thomas et al., 25). Ethical approval for NorStOP was obtained from the North Staffordshire Local Research Ethics Committee. At baseline, all people aged 50 and over registered with six general practices were sent a postal questionnaire containing general health, sociodemographic and pain questions. In the United Kingdom, approximately 98% of people are registered with a general practice for their health care (Bowling et al., 5). The registered populations of these general practices can be assumed to be representative of the wide range of sociodemographic status found in the North Staffordshire area. Questionnaires were sent at baseline, 3 years and 6 years, and the data from these were used to establish three subgroups:

1. Group 1 reported no pain at all three time points.
2. Group 2 reported pain, but no interference from this pain, at all three time points.
3. Group 3 reported pain that interfered with their lives at all three time points.

The measure of pain was based on reporting at least one area of pain that lasted for more than a day. Respondents were asked to shade in the location(s) on a body pain manikin of any pain that had lasted for 1 day or more in any part of their body in the past 4 weeks (Lacey et al., 11). In this context, we have used the term ‘long term’ to reflect consistent reporting at the three time points rather than to imply continuous states. For those shading in at least one area of pain on the manikin, the presence of interfering pain was assessed using one item from the general health status measure, the SF-36: ‘During the past 4 weeks, how much did pain interfere with your normal work (including both work outside the home and housework)?’ (Ware and Sherbourne, 28). Respondents answering ‘moderately’, ‘quite a bit’ or ‘extremely’ on the five-category response scale were defined as having pain that interfered with their lives (Blyth et al., 4; Thomas et al., 24, 23; Jordan et al., 10). This item relates to any pain, is not site specific and does not directly ask about areas shaded on the manikin. The validity and reliability of this item in this population has been established previously (Jordan et al., 10).

### 2.1 Other pain measures

Independently of the manikin and pain interference item, all respondents were asked to report the intensity of their usual pain over the last 6 months for each of 11 specified parts of the body (elbow, neck, shoulder, knee, head, hand, back, chest, abdomen, hip, foot). Responders rating pain intensity of 5 or more [on a 0 (no pain) to 10 (pain as bad as could be) visual analogue scale] were regarded as having high intensity of pain for that location (based on von Korff et al., 27). Using the American College of Rheumatology criteria, widespread pain was determined from the pain manikin as axial pain and pain in at least two contralateral body quadrants (upper and lower segment plus left- and right-sided pain) (Wolfe et al., 29). Self-reported pain medications used in the past 4 weeks, either with or without a prescription, were grouped by an academic general practitioner into simple analgesics (including paracetamol, aspirin, ibuprofen), non-steroidal anti-inflammatory drugs (Naproxen, Diclofenac, Celecoxib, Etoricoxib, Meloxicam, Lumiracoxib), prescription only analgesics (Tramacet, Co-proxamol, Co-dydramol, Tramadol, Dihydrocodeine), complementary therapies (glucosamine, chondroitin sulphate, fish oil, herbal remedies) and topical treatments.

### 2.2 Physical and mental health

Physical function was assessed using the physical functioning scale of the SF-36 version 2 (Ware and Sherbourne, 28). Depression and anxiety were measured using the Hospital Anxiety and Depression Scale; scores of 8 or more indicate possible or probable anxiety or depression (Zigmond and Snaith, 30). There was a high level of agreement between the anxiety and depression scales, with 79% of participants being neither anxious nor depressed, or being both anxious and depressed, at 6 years. Therefore, we combined the two scales.

### 2.3 Sociodemographic and co-morbidity

Sociodemographic and co-morbidity measures previously identified as being linked to disabling pain were also measured. Individual socio-economic factors measured included living arrangement (living alone or not), continuing further education after leaving school, socio-economic status (based on current or last job) (ONS, 14), perceived adequacy of income (‘find it a strain to get by’ or ‘have to be careful with money’ compared with ‘able to manage without difficulty’ or ‘quite comfortably off’) and social networks based on the Berkman–Syme index (Berkman and Syme, 3). The Berkman–Syme index includes items on the number of, and recent contact with, close friends and relatives, as well as church membership and participation in informal and formal groups. Individuals are categorized as having low, medium, medium-high or high social networks. For the purposes of this study, we have classified participants into three groups: low or medium; medium-high or high; and unknown. Owing to missing data, 17% of respondents could not be allocated a social network status at baseline; hence, the inclusion of an unknown category. Neighbourhood deprivation was measured using the Index of Multiple Deprivation (IMD) 2004 (Office of the Deputy Prime Minister, 15). This index is based on Super Output Areas (SOAs), of which there are 32,482 in England, with a mean population of 1500. Individuals are allocated to a SOA based on their postcode. The IMD has an overall score, based on a weighted combination of seven domains (income; employment; health and disability; education, skills and training; barriers to housing and services; living environment; and crime). For each domain and for the combined scale, SOAs are ranked from 1 (most deprived) to 32,482 (least deprived). For the analysis presented in this paper, the overall score and the health domain [previously found to be most highly associated with pain interference (Jordan et al., 10)] were used. The SOAs from which the participants in this study were drawn were categorized into three groups: the least deprived 20%, the most deprived 20% and the remaining 60%. This approach has been used previously (Office of the Deputy Prime Minister, 15; Ashworth et al., 2).

Being overweight or obese was defined as having a body mass index greater than 25 based on self-reported height and weight. Co-morbidity was based on self-reporting one or more of chest or heart problems, diabetes or raised blood pressure. Smoking, dichotomized into current or ex-smoker versus non smoker, and alcohol status (at least once a week vs. less than once a week) were also measured.

### 2.4 Statistical analysis

The three pain groups were compared at 6 years on pain intensity, presence of widespread pain, use of pain medication, physical function and mental health scores, sociodemographic characteristics and co-morbidity factors. Baseline physical function and mental health scores were also compared. The main analysis compared the pain without interference group with the other two groups on the sociodemographic and co-morbidity factors at 6 years using multinomial logistic regression. An initial multilevel model to assess the extent of a clustering effect of people within neighbourhoods showed little variation at level 2 (SOA) compared with variation between people, so all analysis was conducted through a single level multinomial logistic regression. Odds ratios (ORs) and 95% confidence intervals (CIs) are presented. As a sensitivity analysis, to account for missing data on the sociodemographic and co-morbidity covariates in the 6-year questionnaire, multiple imputation (with five imputations) was used. The imputations were based on responses to the relevant variable at baseline and 3 years (where available), age, gender and pain interference status. The sensitivity analysis resulted in data from 1841 of the 1880 participants being used for the multivariable analysis based on the multiple imputation data.

A final subgroup analysis, determined a priori, was restricted to those who reported both widespread pain and at least one high pain intensity site at 6 years. Those with pain that did not interfere at all three time points were compared to those with interfering pain within this subgroup on sociodemographic and co-morbidity factors at 6 years, using binary logistic regression.

Analyses were performed using MLwiN 2.20 (Rasbash et al., 18), PASW Statistics 18.0 and Stata/IC 11.1 for Windows.

## 3. Results

A total of 19,818 people aged 50 and over were sent the initial postal questionnaire at baseline. Responses were received from 13,986 (71%) individuals. At baseline, men and those aged 56–64 were less likely to respond. A total of 4756 people also responded to the 3- and 6-year follow-up surveys. These respondents did not differ significantly in gender from those who did not complete questionnaires at 6 years. However, those responding at 6 years were younger (mean difference 5.2 years; 95% CI 4.8, 5.5) and less likely to have reported pain interference at baseline (difference in percentage reporting pain interference 4.9%; 95% CI 3.2%, 6.6%).

Of these 4756 respondents, 1880 (40%) reported the same level of interference of pain at baseline, 3 years and 6 years. A total of 899 (19% of all respondents; 95% CI 18%, 20%) reported pain that interfered with their lives at all three time points; 560 (12%; 95% CI 11%, 13%) reported pain that did not interfere with their lives at all three time points; and 421 (9%; 95% CI 8%, 10%) reported no pain at all three time points.

Table 1 compares pain intensity, widespread pain and use of pain medications at 6 years between the three groups. Ninety-five percent of those in the ‘pain that interferes’ group reported high pain intensity in the last 6 months in at least one region compared with 59% of the ‘pain without interference’ group and only 17% of the ‘no pain’ group. This ordering across the groups was also evident on examining the number of regions of high pain intensity. Two-thirds of those with interfering pain had widespread pain compared with a third of those with pain that did not interfere. There were fewer clear-cut differences on use of pain medication between these two groups, although the ‘pain with interference’ group was more likely to have used prescription analgesics (61% vs. 16%). Overall, 66% of the ‘no pain’ group, 90% of the ‘pain without interference’ group and 97% of the ‘pain with interference’ group had used at least one pain medication in the previous 4 weeks.

**Table 1:** Pain intensity, widespread pain and use of pain medications at 6 years

	No pain	Non-interfering pain	Pain that interferes
Total	421	560	899
No. of high pain intensity sites^a^; median (IQR)	0 (0,0)	1 (0, 2)	4 (2, 6)
At least one high pain intensity site^a^ *n* (%)	72 (17)	329 (59)	853 (95)
Widespread pain^b^ *n* (%)	0 (0)	186 (33)	589 (66)
No. of pain medications^c^; median (IQR)	1 (0,2)	2 (1, 4)	3 (2,4)
Simple analgesic^d^ *n* (%)	224 (53)	415 (74)	659 (73)
NSAID^d^ *n* (%)	9 (2)	38 (7)	139 (15)
Prescription analgesic^d^ *n* (%)	28 (7)	87 (16)	546 (61)
Complementary medication^d^ *n* (%)	116 (28)	282 (50)	429 (48)
Topical treatment^d^ *n* (%)	37 (9)	171 (31)	419 (47)

All comparison between groups, *P* < 0.001 from chi-squared tests (no. of high pain intensity sites and no. of pain medications: Kruskal–Wallis test). IQR, interquartile range; NSAID, non-steroidal anti-inflammatory drug.

a Pain intensity in last 6 months in 11 sites (headache, neck, shoulder, elbow, hand, chest, abdominal, back, hip, knee, foot); high pain intensity defined as score of 5 or more.

b Based on American College of Rheumatology criteria (Wolfe et al., 29).

c Use of different pain medications (prescribed or over the counter) in previous 4 weeks.

d In past 4 weeks.

The ‘pain without interference’ group was much more similar to the ‘no pain’ group than to the ‘pain with interference’ group on mean physical function, anxiety and depression scores (Table 2). However, they had statistically significantly worse scores on these three measures at both baseline and 6 years than the ‘no pain’ group, except for depression at baseline (*P* = 0.07) and for depression at 6 years (*P* = 0.56). Generally, for all three groups there was a slight worsening of physical functioning score, and improvement in depression and anxiety scores (i.e. reduction in level of depression or anxiety), from baseline to 6 years.

**Table 2:** Mean (standard deviation) physical function, anxiety and depression scores at baseline and 6 years

	No pain	Non-interfering pain	Pain that interferes
	(*n* = 421)	(*n* = 560)	(*n* = 899)
SF36 physical function^a^			
Baseline	89.7 (13.00)	83.5 (13.76)	33.9 (24.10)
6 years	83.1 (20.81)	80.1 (16.69)	27.9 (22.84)
HADS depression score^b^			
Baseline	2.63 (2.64)	2.93 (2.48)	6.73 (3.67)
6 years	2.37 (2.62)	2.47 (2.28)	6.94 (3.78)
HADS anxiety score^b^			
Baseline	4.58 (3.44)	5.73 (3.63)	8.91 (4.40)
6 years	3.53 (3.23)	4.50 (3.14)	8.06 (4.37)

HADS, Hospital Anxiety and Depression Scale.

a Range 0–100, 100 best.

b Range 0–21, 0 best.

Descriptive comparisons of the ‘pain without interference’ group to the other two groups on 6-year sociodemographic and co-morbidity factors are shown in Table 3. Comparison of the ‘pain without interference’ group to the ‘no pain’ group showed few differences other than for age and gender (Table 4). Women were less likely to be in the ‘no pain’ group (adjusted OR 0.65; 95% CI 0.48, 0.87), but those in the oldest age group were more likely to be in the ‘no pain’ group than in the ‘pain without interference’ group (age 80+ compared with age 56–64: OR 2.16; 95% CI 1.24, 3.79).

**Table 3:** Socio-economic factors and co-morbidity at 6 years by pain interference status

	No pain *n* (%)	Non-interfering pain *n* (%)	Pain that interferes *n* (%)
Total	421	560	899
Women	210 (50)	322 (58)	530 (59)
Age at 6 years			
56–64	160 (38)	276 (49)	266 (30)
65–79	203 (48)	252 (45)	494 (55)
80+	58 (14)	32 (6)	139 (15)
Overweight/obese	210 (51)	326 (60)	636 (74)
Current/ex-smoker	193 (46)	288 (52)	509 (57)
Alcohol at least once a week	232 (56)	346 (63)	360 (40)
Living alone	126 (30)	119 (22)	269 (30)
No further education	342 (83)	428 (78)	807 (92)
Income inadequate	118 (30)	172 (31)	496 (60)
Co-morbid^a^	202 (48)	282 (50)	687 (76)
Depressed or anxious^b^	63 (15)	90 (16)	527 (60)
Low/medium social networks	216 (64)	262 (56)	492 (67)
Socio-economic class			
Managerial/professional	98 (23)	165 (29)	128 (14)
Intermediate	96 (23)	113 (20)	162 (18)
Routine	208 (49)	268 (48)	544 (61)
Other	19 (5)	14 (3)	65 (7)
Area deprivation (overall)			
Least deprived	58 (14)	96 (17)	76 (8)
Mid deprived	313 (74)	404 (72)	643 (72)
Most deprived	50 (12)	60 (11)	180 (20)
Area deprivation (health domain)			
Least deprived	51 (12)	79 (14)	62 (7)
Mid deprived	316 (75)	423 (76)	645 (72)
Most deprived	54 (13)	58 (10)	192 (21)

a Chest pain, heart problems, diabetes or raised blood pressure.

b HADS score ≥8 on depression or anxiety scale.

**Table 4:** Comparisons of socio-economic and co-morbidity factors at 6 years between (1) no pain and non-interfering pain groups and (2) pain that interferes and non-interfering pain groups

	(1) No pain	(2) Pain that interferes
	OR^a^ (95% CI)	OR^a^ (95% CI)
Men	1.00	1.00
Women	0.65 (0.48, 0.87)^b^	0.94 (0.70, 1.25)
Age at 6 years		
56–64	1.00	1.00
65–79	1.27 (0.94, 1.70)	2.30 (1.72, 3.08)^b^
80+	2.16 (1.24, 3.79)^b^	5.12 (2.98, 8.81)^b^
Not overweight/obese	1.00	1.00
Overweight/obese	0.83 (0.62, 1.10)	2.25 (1.67, 3.03)^b^
Never smoked	1.00	1.00
Previous/current smoker	0.75 (0.57, 1.00)	1.20 (0.91, 1.60)
Alcohol less than once a week	1.00	1.00
Alcohol at least once a week	0.81 (0.60, 1.08)	0.52 (0.39, 0.69)^b^
Not living alone	1.00	1.00
Living alone	1.35 (0.96, 1.91)	1.13 (0.80, 1.58)
Further education	1.00	1.00
No further education	1.10 (0.76, 1.60)	1.77 (1.18, 2.68)^b^
Income not inadequate	1.00	1.00
Income inadequate	0.86 (0.63, 1.17)	2.05 (1.55, 2.71)^b^
No co-morbidity	1.00	1.00
Co-morbidity^c^	0.85 (0.64, 1.12)	1.86 (1.41, 2.47)^b^
Not depressed or anxious	1.00	1.00
Depressed or anxious^d^	1.02 (0.70, 1.49)	6.36 (4.67, 8.65)^b^
Medium-high/high networks	1.00	1.00
Low/medium networks	1.22 (0.88, 1.69)	1.00 (0.73, 1.38)
Unknown networks	1.18 (0.78, 1.79)	0.86 (0.57, 1.29)
Socio-economic class		
Managerial/professional	1.00	1.00
Intermediate	1.26 (0.84, 1.90)	1.32 (0.87, 2.01)
Routine	1.16 (0.81, 1.65)	1.49 (1.04, 2.14)^b^
Other	1.50 (0.65, 3.48)	3.21 (1.53, 6.70)^b^
Area deprivation – overall		
Least deprived	1.00	1.00
Mid deprived	1.35 (0.91, 1.99)	1.61 (1.06, 2.44)^b^
Most deprived	1.25 (0.72, 2.18)	1.45 (0.84, 2.49)

CI, confidence interval; OR, odds ratio.

a The ‘Non-interfering pain’ group is the reference category for all between-group comparisons. Adjusted for the other presented variables.

b Statistically significant at *P* < 0.05.

c Chest pain, heart problems, diabetes or raised blood pressure.

d HADS score ≥8 on depression or anxiety scale.

By contrast, the ‘pain without interference’ group was distinct from those with pain that interfered with life. Those with interference from pain were more likely to report depression or anxiety (adjusted OR 6.36; 95% CI 4.67, 8.65), be overweight or obese (OR 2.25; 95% CI 1.67, 3.03), report inadequate income (OR 2.05; 95% CI 1.55, 2.71) and be more likely to be in the older age groups (age 80+ compared with age 56–64: OR 5.12; 95% CI 2.98, 8.81). The ‘pain with interference’ group was also more likely, but with less strong associations, to report a co-morbidity, to have not attended further education and to be of lower social class. There was also a link with neighbourhood deprivation, with those in the ‘pain with interference’ group more likely to come from a more deprived area, particularly in terms of health deprivation (compared with least deprived areas: OR 1.68; 95% CI 0.95, 2.98). However, they were less likely to drink alcohol at least once a week (OR 0.52; 95% CI 0.39, 0.69).

A total of 126 (23%) of the ‘pain without interference’ group and 572 (64%) of the ‘pain with interference’ group reported widespread pain and at least one high intensity pain site. Comparisons between these two subgroups yielded associations of similar magnitude to those derived from the main analysis (Table 5). The exception was that neighbourhood deprivation was no longer distinct between the two groups.

**Table 5:** Associations of socio-economic and co-morbidity factors at 6 years with pain interference status adjusted for other presented variables in those with widespread pain and at least one high pain intensity site

	Non-interfering pain *n* (%)	Pain that interferes *n* (%)	OR^a^ (95% CI)
Total	126	572	
Men	51 (40)	221 (39)	1.00
Women	75 (60)	351 (61)	1.03 (0.61, 1.73)
Age at 6 years			
56–64	69 (55)	192 (34)	1.00
65–79	51 (40)	309 (54)	2.63 (1.59, 4.37)^b^
80+	6 (5)	71 (12)	6.27 (2.03, 19.31)^b^
Not overweight/obese	45 (36)	130 (24)	1.00
Overweight/obese	79 (64)	422 (76)	3.34 (1.95, 5.72)^b^
Never smoked	64 (51)	247 (43)	1.00
Previous/current smoker	62 (49)	322 (57)	1.09 (0.67, 1.78)
Alcohol less than once a week	56 (44)	342 (60)	1.00
Alcohol at least once a week	70 (56)	225 (40)	0.75 (0.45, 1.23)
Not living alone	98 (78)	410 (72)	1.00
Living alone	28 (22)	157 (28)	1.43 (0.77, 2.67)
Further education	28 (22)	47 (8)	1.00
No further education	97 (78)	512 (92)	2.09 (1.06, 4.14)^b^
Income not inadequate	72 (59)	210 (39)	1.00
Income inadequate	51 (41)	326 (61)	1.58 (0.96, 2.59)
No co-morbidity	57 (45)	141 (25)	1.00
Co-morbidity^c^	69 (55)	431 (75)	1.66 (1.03, 2.70)^b^
Not depressed or anxious	101 (81)	214 (38)	1.00
Depressed or anxious^d^	24 (19)	347 (62)	6.97 (4.02, 12.08)^b^
Medium-high/high networks	45 (36)	158 (28)	1.00
Low/medium networks	59 (47)	311 (54)	0.93 (0.53, 1.62)
Unknown networks	22 (17)	103 (18)	0.87 (0.43, 1.75)
Socio-economic class			
Managerial/professional	31 (25)	80 (14)	1.00
Intermediate	29 (23)	99 (17)	1.05 (0.50, 2.20)
Routine	63 (50)	357 (62)	1.49 (0.78, 2.84)
Other	3 (2)	36 (6)	3.25 (0.83, 12.76)
Area deprivation – overall			
Least deprived	13 (10)	43 (8)	1.00
Mid deprived	97 (77)	415 (73)	1.32 (0.57, 3.04)
Most deprived	16 (13)	114 (20)	0.90 (0.33, 2.46)

CI, confidence interval; OR, odds ratio.

a The ‘Non-interfering pain’ group is the reference category.

b Statistically significant at *P* < 0.05.

c Chest pain, heart problems, diabetes or raised blood pressure.

d HADS score ≥8 on depression or anxiety scale.

Analysis based on multiple imputation data yielded very similar estimates and CIs to those from the complete case analysis presented above.

## 4. Discussion and conclusions

Unchanging pain status is common in older people, with 40% of our surveyed population reporting consistent pain status at all three time points over 6 years. Our study also found that pain that is non-interfering, in that it does not interfere in everyday activities, can be a long-term status. Those with non-interfering pain were similar to those with no pain when comparing measures based on social factors and co-morbidity. However, it is evident that individuals within the non-interfering pain group can have high levels of pain: over one-half reported high pain intensity over the previous 6 months in at least one body region; one-third also had widespread pain; and 90% had used pain medication in the past 4 weeks. However, the physical and mental self-reported health of this group was generally stable over 6 years.

The group with non-interfering pain differed extensively from the group with no pain only in relation to the fact that they were more likely to be female and to be younger. Some of this younger group may go on to develop disabling pain as they age, and future longitudinal research could usefully investigate this.

This study has shown that there are distinct co-morbid and socio-economic differences between those with chronic interfering pain and those with chronic non-interfering pain. Those with interfering pain were more likely to be depressed, overweight, have inadequate income, be in the older age group, have co-morbidities, have lower levels of education and reside in a more deprived area. These differences remained when restricting the analysis to those with both widespread and high intensity pain, suggesting that the differences are not due simply to having more widespread or more severe pain. The emergence of social factors such as adequacy of income and neighbourhood deprivation again highlights their importance in studies of interfering pain, as shown previously (Jordan et al., 10; Shi et al., 20). Previous studies have investigated links between fewer social relationships and the reporting of pain that interferes with life, with mixed results (Jakobsson et al., 9; Peat et al., 16). Among those reporting pain, Peat and colleagues showed that the absence of social ties and of contact with close friends was associated with having pain that interfered with daily life (Peat et al., 16). However, this relationship was weakened after adjusting for sociodemographic variables such as employment and after adjusting specifically for depression and co-morbidity, although there was still a relationship with extent of contact with one's children. In our study of people with long-term unchanging pain status, social networks did not appear to be related to their long-term pain status, and hence may be more related to changing pain status.

As non-interfering pain has previously been shown to be the strongest independent predictor of pain that does interfere with life (Jordan et al., 10), this suggests that those with long-term non-interfering pain should be targeted in further research, to assess how the development of interfering pain could be controlled. A further qualitative phase to our study utilizes in-depth interviews with those who reported the same pain status (no pain, non-interfering pain, interfering pain) at each of the three time points. It explores individuals' own identification and interpretation of the factors they believe are relevant to their past and present experience of pain. Informed by the individual's survey data and GP medical and prescription data, the interviews enable a fuller exploration of demographic, cultural and psychosocial factors. This phase hence examines the relationship between the quantitative associations with pain status that we have reported and individuals' own beliefs and perceptions of the causes and how they self-manage their pain.

The question used in this study relating to pain interference reflects respondents' viewpoints about the extent of disruption to their own life that they attribute to pain and has a strong association with more extensive or specific measures of disability. However, it is a general question, in that it is not specific to any particular pain and, furthermore, may be interpreted differently by different respondents (Adamson et al., 1; Smith, 21). The notion of ‘interference’ may be different for those who have redefined their vocabulary in an attempt to gain control of their pain or who have modified the ways in which they carry out their daily activities. It is possible that some of those who noted that they had ‘no interference’ from their pain may have defined ‘interference’ as something that was beyond their control, and may therefore have discounted it in responding to this question. Our qualitative explorations also assess whether, through acceptance of the pain and its limitations, these individuals may have reorganized and reassessed their goals and adjusted their expectations, thereby regaining control of their lives, and no longer perceive their pain as interfering with their daily activities (McCracken and Eccleston, 13; McCracken et al., 12).

Lessons for secondary prevention may then emerge from the combined quantitative and qualitative work in terms of effective self-management to prevent pain interfering with life that may be utilized in public health promotion initiatives.

While based on a large cohort study, the analysis reported here is essentially cross-sectional and therefore temporal relationships cannot be established. There was attrition at each stage of the study and differences in response by sociodemographic status may affect the estimates of the older population who have stable pain status within the three groups, although these differences should not affect the associations reported. Definitions of pain and disability were based solely on the previous 4 weeks at all three time points. Therefore, exacerbations and recurrences over the 6 years would not be captured. Pain may also in some cases reflect episodes of acute pain as the definition was pain lasting at least 1 day. The ‘no pain’ group had used pain medication, particularly basic analgesia, but given that our definition of pain was that it should last longer than a day, their pain was likely to be short-lasting. However, it is possible that some of this group effectively managed longer term pain using simple pain medications. The measure of co-morbidity was limited and it is possible that a more comprehensive measure would have identified a stronger relationship with pain status. Finally, there may be other factors that are distinguishable between the groups, but which were not measured here.

This study has highlighted the importance of studying the extent of interference from pain rather than focusing solely on the presence of pain. Pain that is non-interfering can be a long-term state, characterized by high levels of pain medication and frequently involving widespread pain. However, despite this, those with non-interfering pain do not have many of the characteristics associated with interfering pain and appear to be able to control the effects of their pain. Future work will assess how people in this group control their pain to ensure it does not cause interference with their lives.
